# NUF2 promotes tumorigenesis by interacting with HNRNPA2B1 via PI3K/AKT/mTOR pathway in ovarian cancer

**DOI:** 10.1186/s13048-023-01101-9

**Published:** 2023-01-20

**Authors:** Meng Ren, Hongyu Zhao, Yan Gao, Qi Chen, Xiaoting Zhao, Wentao Yue

**Affiliations:** 1 Central Laboratory, Beijing Obstetrics and Gynecology Hospital, Capital Medical University, Beijing Maternal and Child Health Care Hospital, Beijing, 100026 People’s Republic of China; 2 Center for Discovery and Innovation, Hackensack Meridian Health, Nutley, NJ 07110 USA

**Keywords:** NUF2, Ovarian cancer, HNRNPA2B1, PI3K/AKT/mTOR signaling pathway

## Abstract

**Background:**

Ovarian cancer (OC) is one of the commonest and deadliest diseases that threaten the health of women worldwide. It is essential to find out its pathogenic mechanisms and therapeutic targets for OC patients. Although NUF2 (Ndc80 kinetochore complex component) has been suggested to play an important role in the development of many cancers, but little is known about its function and the roles of proteins that regulate NUF2 in OC. This study aimed to investigate the effect of NUF2 on the tumorigenicity of OC and the activities of proteins that interact with NUF2.

**Methods:**

Oncomine database and immunohistochemical (IHC) staining were used to evaluate the expression of NUF2 in OC tissues and normal tissues respectively. Normal ovarian epithelial cell lines (HOSEpiC) and OC cell lines (OVCAR3、HEY、SKOV3) were cultured. Western blot was applied to analyze the expression of NUF2 in these cell lines. Small interfering RNA (siRNA) was used to silence the expression of NUF2 in OC cell lines, SKOV3 and HEY. Gene Set Variation Analysis (GSVA), Gene Set Enrichment Analysis (GSEA), the CCK-8 method, colony formation assay and flow cytometry were conducted to analyze the biological functions of NUF2 in vitro. OC subcutaneous xenograft tumor models were used for in vivo tests. Immunoprecipitation and mass spectrometry (IP/MS) were performed to verify the molecular mechanisms of NUF2 in OC. IP, immunofluorescence, IHC staining, and Gene Expression Profiling Interactive Analysis platform (GEPIA) were used to analyze the relationship between HNRNPA2B1 and NUF2 in OC cells. SiRNA was used to silence the expression of HNRNPA2B1 in SKOV3 cells, reverse transcription quantitative polymerase chain reaction (RT-qPCR) assay and western blot were used to detect the effect of HNRNPA2B1 on NUF2. GEPIA, The Cancer Genome Atlas (TCGA) database, GSEA and western blot were used to detect the potential signaling pathways related to the roles of HNRNPA2B1 and NUF2 in OC cells.

**Results:**

Our results showed high NUF2 expression in OC tissues and OC cell lines, which was associated with shorter overall survival and progression-free survival in patients. NUF2 depletion by siRNA suppressed the proliferation abilities and induced cell apoptosis of OC cells in vitro, and impeded OC growth in vivo. Mechanistically, NUF2 interacted with HNRNPA2B1 and activated the PI3K/AKT/mTOR signaling pathway in OC cells.

**Conclusion:**

NUF2 could serve as a prognostic biomarker, and regulated the carcinogenesis and progression of OC. Moreover, NUF2 may interact with HNRNPA2B1 by activating the PI3K/AKT/mTOR signaling pathway to promote the development of OC cells. Our present study supported the key role of NUF2 in OC and suggested its potential as a novel therapeutic target.

**Supplementary Information:**

The online version contains supplementary material available at 10.1186/s13048-023-01101-9.

## Introduction

Ovarian cancer (OC) is the deadliest malignant tumor among female reproductive system diseases, and its mortality rate ranks fifth among all female malignancies [1]. High-grade serous ovarian cancer (HGSOC) is the most common and aggressive type of OC [2]. Currently, the standard treatment for OC is surgery combined with chemotherapy. Despite this, the 5-year survival rate of the patients is only 45% [3]. However, the pathogenesis of OC is not yet fully defined. Therefore, it is extremely important to deeply study the underlying molecular pathogenesis of OC in order to identify new therapeutic targets.

Dysfunction of chromosome segregation during the mitosis process is generally considered one of the important reasons that contribute to cancer progression. The kinetochore is the protein that connect the spindle and the centromere in mitotic cells [4]. The outer kinetochore is critical for accurate chromosome segregation. As a core component of the outer kinetochore, the NDC80 complex is essential for regulating chromosome segregation. NUF2, one of the components of NDC80 kinetochore complex, which has been suggested to be essential for kinetochore microtubule formation. Knockout of NUF2 results in abnormal kinetochore-microtubule attachment and spindle inspection, leads to the death of cells [5]. There are mounting evidences that NUF2, as an oncogene, is closely related to the growth, apoptosis, invasion and metastasis of various tumor cells and poor prognosis of cancer patients [6–11]. However, the biological roles of NUF2 and the underlying molecular mechanisms of its actions in OC are still not fully elucidated.

In this study, we identified high expression of NUF2 in OC tissues and OC cell lines. In addition, in vitro and in vivo experiments indicated that knockdown of NUF2 suppressed OC cell proliferation, and promoted cell apoptosis by inactivated the PI3K/AKT/mTOR signaling pathway. Moreover, we found HNRNPA2B1, which had been previously validated regulating the carcinogenesis and progression of OC [12], could interact with and regulate NUF2. HNRNPA2B1 or NUF2 downregulation by siRNA significantly blocked the PI3K/AKT/mTOR signaling pathway. Overall, our findings indicated that the interaction between HNRNPA2B1 and NUF2 promoted the progression of OC by influencing PI3K/AKT/mTOR pathway.

## Materials and methods

### Database analysis

The mRNA expression levels of NUF2 in OC and normal ovarian tissues were downloaded from the Oncomine database (http://www.oncomine.org). The Kaplan–Meier plotter database (http://www.kmplot.com) was used to explore the prognosis of 1657 OC patients. The OC patients were divided into two groups according to the expression of the gene (high expression vs low expression). RNA-seq data and corresponding clinicopathological data of OC patients in TCGA were obtained from UCSC Xena (https://xenabrowser.net/datapages/). The clinicopathological characteristics included age, stage, grade, outcome, etc. A GSEA was conducted based on protocols according to the manufacturer’s instructions to identify the NUF2-mediated biological roles in OC [13]. Specifically, GSEA was conducted by clusterProfiler package. We downloaded h.all.v6.2.symbols.gmt from the Molecular Signatures Database (http://www.broad.mit.edu/gsea/msigdb/). The number of permutations was set to 1,000 and the phenotype labels were NUF2‑high and NUF2‑low. FDR < 0.25 and *P* < 0.05 indicated statistical significance. 309 OC patients in the TCGA data set were divided into high-expression group and low-expression group according to the median value of NUF2. GSVA was conducted by “gsva” package to further verify different biological parameters between the two groups. Besides, GSEA of enriched pathways was conducted in 379 OC patients exhibited high HNRNPA2B1 expression or low HNRNPA2B1 expression grouped by median value of HNRNPA2B1. In addition, the GEPIA (http://gepia.cancer-pku.cn/) platform was used to explore the relationships among NUF2 expression, HNRNPA2B1 expression and molecules involved in the PI3K/AKT/mTOR signaling pathway.

### Patients and samples

HGSOC tissues (*n* = 13) and matched adjacent tissues (*n* = 13) were collected from the Biobank of Beijing Obstetrics and Gynecology Hospital. Samples were obtained from patients at initial diagnosis without any chemotherapy or radiation treatment prior to surgery. The present study was approved by medical ethics association of Beijing Obstetrics and Gynecology Hospital, Capital Medical University. Informed consents were obtained from all HGSOC patients.

### Immunohistochemistry

All of the tissues were fixed in 4% paraformaldehyde, embedded in paraffin, and then serially sectioned. The sections were treated with primary antibodies (Cell Signaling Technology, Inc., Danvers, MA, USA) against NUF2 (1:200 dilution), HNRNPA2B1 (1:200 dilution) respectively overnight in a humidified chamber at 4 °C. Afterwards, the sections were incubated with secondary antibodies. Sections were then stained with 3,3diaminobenzidine (DAB) and counterstained with hematoxylin. Two pathologists calculated the staining score of each section independently. The immunoreaction intensity was scored as follows: negative (0), weak (1), moderate (2), strong (3). The percentage of stained cells was as follows: 1–25% (1); 26–50% (2), 51–75% (3) and 76–100% (4). The staining score of ≥ 2 was denoted high protein expression.

### Cell culture

The Human Ovarian Surface Epithelial Cells (HOSEpiC) and three human OC cell lines (OVCAR3, HEY, SKOV3) were maintained in laboratory. The OVCAR3 was cultured in Roswell Park Memorial Institute (RPMI)-1640 medium (Gibco, Gaithersburg, MD, USA), supplemented with 20% fetal bovine serum (FBS). All other cancer cell lines were cultured in RPMI-1640 medium, supplemented with 10% FBS. All cell lines were cultured in a humidified incubator with 5% CO2 at 37ºC.

### Small interference RNA and cells transfection

The siRNA-mediated silencing system (JTSBIO Co., Ltd. Wuhan, China) targeting NUF2 contained three siRNAs: siRNA1: 5'-GGAGAGACUUGAUUCUGUUTT-3'; siRNA2: 5'-GCCUUCAUGUCAGUUGGAATT-3' and siRNA3: 5'-GGACCAAAUUGAGAGUGAUTT-3'. The siRNA-mediated silencing system (JTSBIO Co., Ltd. Wuhan, China) targeting HNRNPA2B1 contained three siRNAs: siRNA1: 5'-GCUCUUUAUUGGUGGCUUAT-3'; siRNA2: 5'- GGAACAUCACCUUAGAGAUTT-3' and siRNA3: 5'- GGUCAUAAUGCAGAAGUAATT-3'. The sequence of nonsense siRNA (si-control) is 5'-UUCUCCGAACGUGUCAGGUTTUCCAGGTCUAGTT-3'. Si-control, si-HNRNPA2B1 and si-NUF2 were transfected into HEY and SKOV3 cells cultured in 6-well plates at 2 × 10^5^ cells/well respectively as si-control group, si-NUF2 group and si-HNRNPA2B1 group by using Lipofectamine™ RNAmax. After 48 h of transfection, the transfection efficiency was verified by Western blot.

### Western blot

Proteins were extracted from HEY and SKOV3 cells using lysis buffer. 30 µg of protein was loaded onto the SDS-PAGE gel and then transferred onto the polyvinylidene difluoride membranes (PVDF) membranes. These membranes were blocked with 5% milk and then incubated overnight at 4 °C with primary antibodies (Cell Signaling Technology, Inc., Danvers, MA, USA) against NUF2 (dilution, 1:500), caspase3 (dilution, 1:500), cleaved-caspase3 (dilution, 1:500), PARP (dilution, 1:500), cleaved-PARP (dilution, 1:500), PIK3CA (dilution, 1:500), PIK3CB (dilution, 1:500), AKT (dilution, 1:500), p-AKT (dilution, 1:500), mTOR (dilution, 1:500), p-mTOR (dilution, 1:500), and GAPDH (dilution, 1:1000). Then the membranes were incubated with HRP-labeled secondary antibodies (dilution, 1:7500; Cell Signaling Technology, Inc., Danvers, MA, USA) for 2 h at room temperature. Enhanced chemiluminescence system (Roche Diagnostics, Basel, Switzerland) was used to detect the target bands. The intensities of the bands were quantified using Image J software. All of the experiments were technically repeated three times.

### Proliferation assay

Cell counting kit-8 (CCK-8) assay and colony formation assay were used to detect the proliferation of cells. For CCK-8 assay, cell counting kit-8 assay kit (Yeasen Biotech Co., Ltd. Shanghai, China) was used. Cells transfected with si-NUF2 or si-control were seeded in a 96-well plate at a density of 1 × 10^3^ cells/ml with three duplicates in each group. After cultured for 24, 48, 72, and 96 h, respectively, the supernatant was discarded, 10 μl of CCK8 reagent was added to each well and incubated at 37 °C in an incubator for 2 h. Measure the optical density of each well at 450 nm with a microplate reader. For colony formation assay, 1500 transfected cells were seeded into 6-well plate and incubated for 5 days in a humidified incubator. Then the clones were fixed by 4% paraformaldehyde and stained for 15 min in Gentian violet. The clones and number of cells in each clone were counted by using a microscope.

### Cell apoptosis analysis

FITC Annexin V Apoptosis Detection Kit (BD, USA) was used to detect cell apoptosis. Cell apoptosis was examined at 48 h after siRNA transfection. Wash cells twice with cold PBS and then resuspend cells in Binding Buffer at a concentration of 1 × 10^6^ cells/ml. Transfer 100 μl of the solution to a 5 ml culture tube. Add 5 μl of FITC Annexin V and 5 μl PI to the solution. Gently vortex the cells and incubate for 15 min at room temperature in the dark. Then, add 400 μl of Binding Buffer to each tube. Finally, analyze cell apoptosis by flow cytometry (FACSCalibur, BD Biosciences, NJ USA).

### Xenograft tumor model

SPF-grade BALB/c-nu mice (5 weeks of age) were purchased from Beijing Weitong Lihua Experimental Animal Technology Co., Ltd. All animal experiments were approved by Institutional Animal Care and Use Committee (IACUC) of Tsinghua University. 5 × 10^6^ SKOV3 cells were injected into the right flanks of the nude mice. When the subcutaneous tumors reached approximately 60 mm^3^ in size, mice were randomized for different intratumoral treatments and treated with PBS (control), scrambled siRNA (si-control) or modified si-NUF2 (si-NUF2). Mice were injected with PBS or the appropriate siRNA (1.5 nM) every 3 days for 2 weeks. Tumor growth was measured using a digital caliper and tumor volume was calculated as follows: tumor volume = (a × b^2^)/2, where a and b are the long and short tumor dimensions respectively. Mice were sacrificed 17 days from the first day of treatment and the tumors were excised.

### IP and Mass spectrometry

SKOV3 cells were lysed in lysis buffer and centrifuged for 10 min at 10000 g. The supernatants were incubated with relevant antibodies and protein A/G beads at 4 °C overnight. Afterwards, SDS-PAGE was performed. The immunocomplex samples were analyzed by Western blot using anti-NUF2 and anti-HNRNPA2B1. For mass spectrometry analysis, the protein bands were obtained from gels and then analyzed by liquid chromatography coupled with tandem Q-Exactive Orbitrap mass spectrometer (UHPLC-QE-MS). UHPLC-QE-MS analysis was conducted by Tsinghua University instrument sharing platform. NCBI protein database was used for data analysis and protein identification.

### Immunofluorescence

SKOV3 cells were fixed in pre-chilled 4% paraformaldehyde in PBS for 20 min, permeabilized with 0.1% Triton X-100 in PBS for 10 min and blocked with 10% normal goat serum in Tris-buffered saline (TBS) containing 2.5% Tween-20 for 1 h at room temperature, then incubated with rabbit anti-NUF2 or rabbit anti-HNRNPA2B1 in the blocking buffer overnight at 4ºC. Rinsing with washing solution (TBS containing 2.5% Tween-20) 3 times, fluorescence-conjugated secondary antibodies (Solarbio, Beijing) were applied for 1 h at room temperature. 4, 6-diamidino-2-phenylindole (DAPI; Invitrogen) was used to stain the nuclei. The staining results were examined by a laser-scanning confocal microscope (FV-1000 mounted on IX81, Olympus).

### RT-qPCR

Complementary DNAs was synthesized using First-strand cDNA Synthesis Supermix kit (TRANS, Beijing, China) according to the manufacturer’ s protocol. ABI 7500 Real-Time PCR system (Applied Biosystems, Foster City, USA) was used for Real-time PCR analysis using SYBR Premix EX Taq™ (Takara, Dalian, China). Relative mRNA expressions were normalized to GAPDH. The primers for NUF2, HNRNPA2B1 and GAPDH were as follows:

- NUF2 forward primer: 5'-ACAAGTCGGTTTTTAAGTGGCA-3', and reverse primer: 5'-GCATTTTGTCCGCAGAGGATTT-3';
- HNRNPA2B1 forward primer: 5'-TGGAGGTAGCCCCGGTTATG-3', and reverse primer: 5'-GGACCGTAGTTAGAAGGTTGCT-3';
- GAPDH forward primer: 5'-TCAACGACCACTTTGTCAAGCTCA-3', and reverse primer: 5' -GCTGGTGGTCCAGGGGTCTTACT-3'.

## Results

### High NUF2 expression in OC and associates with poor survival prognosis

**Fig. 1:**
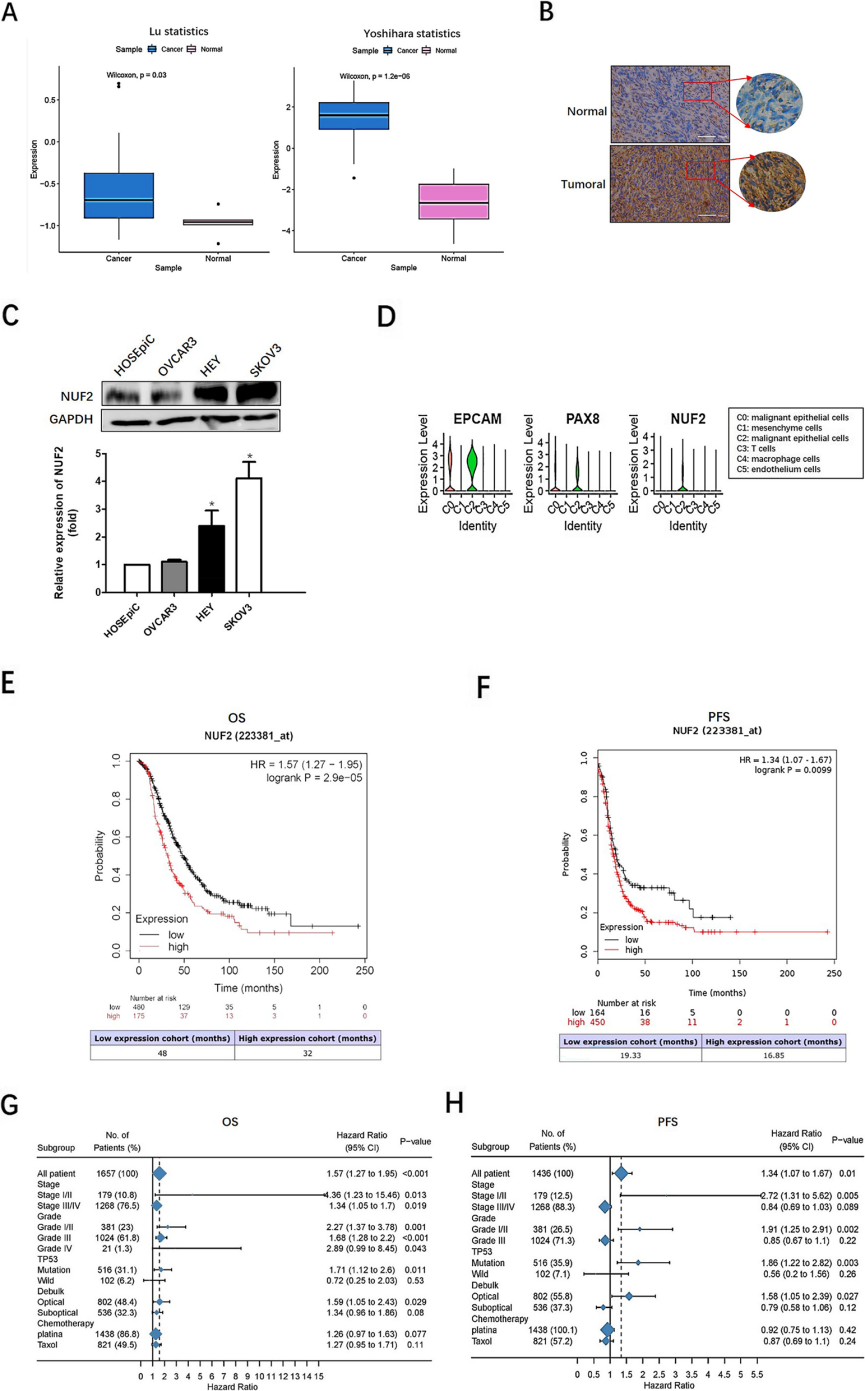
Up-regulation of NUF2 was associated with poor survival in OC. **A** High NUF2 expression was found in OC patients based on oncomine datasets. **B** Immunohistochemical evaluation of NUF2 expression in HGSOC tissues and adjacent normal tissues. Morderate or strong NUF2 expression was confirmed in HGSOC tissues, and negative or weak expression in adjacent normal tissues. Scale bars, 500 μm. **C** The expression levels of NUF2 in OC cell lines were analyzed by western blot. Quantification of NUF2 expression relative to GAPDH were performed. The experiment was repeated 3 times. In each time, the levels of NUF2 relative to GAPDH were normalized to the Human Ovarian Surface Epithelial Cells (HOSEpiC) group that was arbitrarily set as 1. Values are expressed as the mean ± SE. 2-tailed Student t test was used for C. NUF2 protein was overexpressed in HEY and SKOV3 cells compared with Human Ovarian Surface Epithelial Cells (HOSEpiC). *P < 0.05. **D** ScRNA-seq profiling of four primary carcinomas, two relapsed, and two metastatic HGSOCs indicating higher NUF2 expression in malignant epithelial cells (C0 and C2) than other cell types. **E**, **F** Kaplan–Meier (KM) plots indicated the correlation of NUF2 expression with overall survival (OS) and progression-free survival (PFS) of OC patients. Patients with higher NUF2 had poorer OS and PFS. **G**, **H** Subgroup analyses of survival associated with NUF2 in OC patients

To explore the expression of NUF2 in OC, we extracted the data of OC patients and normal patients from Oncomine database, the mRNA level of NUF2 was analyzed. The results showed that NUF2 mRNA levels in OC tissues were higher than that in normal tissues (Fig. 1A). In addition, the expression of NUF2 was analyzed by IHC staining in 13 paired HGSOC tissues and corresponding normal tissues. As shown in Fig. 1B, 77% (10/13) of patients NUF2 staining was significantly increased in HGSOC tissues compared to adjacent normal tissues. Besides, the expressions of NUF2 in OC cell lines were examined by western blotting. As shown in Fig. 1C, overexpression of NUF2 was observed in HEY and SKOV3 cells compared to the HOSEpiC cells at protein level.

To further analyze the prognostic value of NUF2 expression in OC, single cell RNA-sequencing were conducted as previously described [14]. Four primary carcinomas, two metastatic carcinomas, and two recurrent carcinomas were analyzed by deep single-cell RNA sequencing. A total of 21,212 cells were acquired and classified into six main cell lineages, namely, C0-C5. As shown in Fig. 1D, we demonstrated that the expression of NUF2 was significantly higher in malignant epithelial cells (C0 and C2) than the other cells (C1, C3 and C5). Furthermore, Kaplan–Meier plotter database was used to further analysis the clinical value of NUF2 in OC patients. Figure 1E-F demonstrated that high NUF2 expression was associated with shorter overall survival (OS) and progression‑free survival (PFS) compared with low NUF2 expression (*P* < 0.05). Besides, subgroup analyses showed that the OC patients with higher NUF2 presented poorer survival at the early stage or grade (Fig. 1G-H). These results indicated that NUF2 might be an important prognostic factor for OC.

### Related potential biological roles of NUF2 in OC

**Fig. 2:**
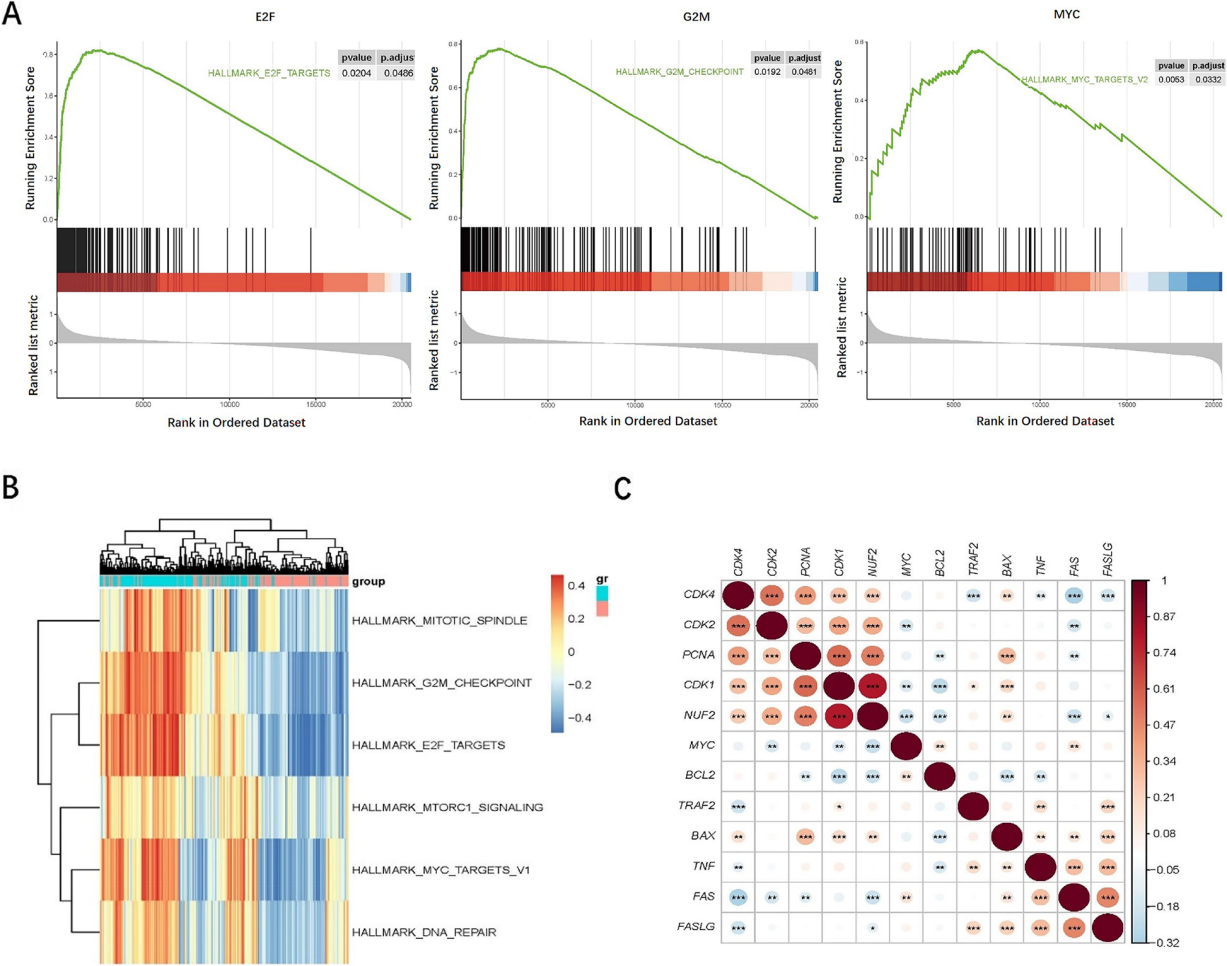
Analysis of enriched pathways of NUF2 using GSVA and GSEA. **A** Enriched pathway found by the GSEA using MsigDB: E2F Targets, G2M Checkpoint, MYC Targets were enriched in OC samples with highly expressed NUF2. **B** Enriched pathway plot found by GSVA. **C** Correlated heatmap illustrated that NUF2 was associated with genes related to the cell cycle, proliferation, and apoptosis

To investigate NUF2-mediated biological roles in OC, the GSEA and GSVA were conducted. GSEA showed that the OC samples with highly expressed NUF2 were mainly associated with E2F targets, G2M checkpoint and MYC signaling, which were confirmed by GSVA (Fig. 2A-B). In addition, correlated heatmap demonstrated that NUF2 expression were positively associated with the cell cycle and proliferation related genes, while negatively associated with the apoptosis related genes in OC (Fig. 2C). These results demonstrated that NUF2 might be related to OC carcinogenesis.

### Silencing of NUF2 expression inhibits proliferation and facilitates apoptosis of OC cell

**Fig. 3:**
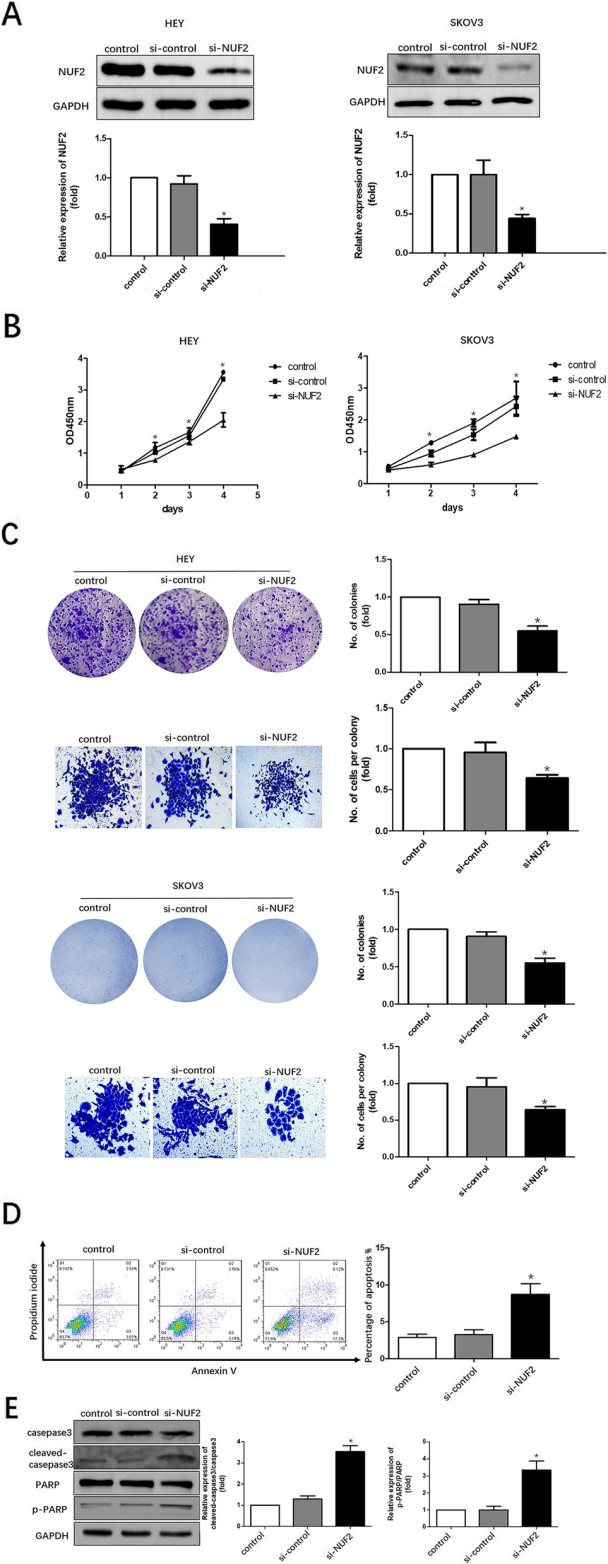
Effect of NUF2 silencing on OC cell proliferation and apoptosis. **A** Western blot was used to validate the efficiency of NUF2 knockdown by small interfering RNA (siRNA) treatment. Quantification of NUF2 expression relative to GAPDH were performed. The result was obtained with 3 independent experiments. In each experiment, the protein levels of NUF2 related to GAPDH were normalized to control group that was arbitrarily set as 1. *P < 0.05. **B** The CCK8 assay was conducted to detect the proliferation of the HEY and SKOV3 cells treated with si-control or si-NUF2 respectively, and knockdown of NUF2 significantly impaired the proliferation of cells. *P < 0.05. **C** Colony formation was conducted to detect the viability of the HEY and SKOV3 cells treated with si-control or si-NUF2 respectively, and knockdown of NUF2 significantly impaired the proliferation of cells. The no. of colonies or no. of cells per colony measured in three independent experiments were statistically analyzed. In each experiment, the no. of colonies or no. of cells per colony were normalized to control group that was arbitrarily set as 1. *P < 0.05. **D** Flow cytometry assay was used to detect the apoptosis of SKOV3 cells treated with si-control or si-NUF2. The result was obtained with 3 independent experiments. Values are expressed as the mean ± SE. 2-tailed Student t test was used for D. **E** Western blot analysis for proteins involved in apoptosis revealed that cleaved-caspase3 and cleaved-PARP were considerably increased after siRNA treatment. The total protein expressions of caspase3 and PARP remained unchanged. Experiments were repeated three times. In each experiment, the protein levels of cleaved-caspase3 or cleaved-PARP related to the total caspase3 or PARP were normalized to the control group that was arbitrarily set as 1

To further explore the potential effect of NUF2 in OC progression, we designed three siRNAs that can silence NUF2 (si-NUF2) and transfected them into HEY and SKOV3 cells respectively to knockdown the expression of NUF2. Cells transfected with control siRNA (si-control) were used as controls. Then western blot was applied to verify the transfection efficiency. As shown in Fig. 3A, the protein levels of NUF2 in HEY and SKOV3 cells transfected with si-NUF2 were significantly lower than those transfected with si-control. These results above indicated that si-NUF2 significantly silenced the expression of NUF2. Furthermore, the effects of silencing NUF2 expression on proliferation of SKOV3 and HEY cells were assessed by CCK-8 assay and colony formation respectively. It was found that silencing NUF2 expression significantly decreased the proliferation of HEY and SKOV3 cells (Fig. 3B-C). Besides, the apoptosis was further confirmed by flow assay. As shown in Fig. 3D, the apoptotic cell percentage increased significantly from 15.7% in si-control group to 27.6% in si-NUF2 group. Furthermore, western blot was used to detect the expression levels of caspase 3, cleaved-caspase3, PARP and cleaved-PARP. We found the increased expressions of cleaved-caspase3, cleaved-PARP after NUF2 silencing (Fig. 3E). These findings indicated that silencing NUF2 expression could induce apoptosis of SKOV3 cells.

### Silencing of NUF2 expression attenuates OC progression in vivo

**Fig. 4:**
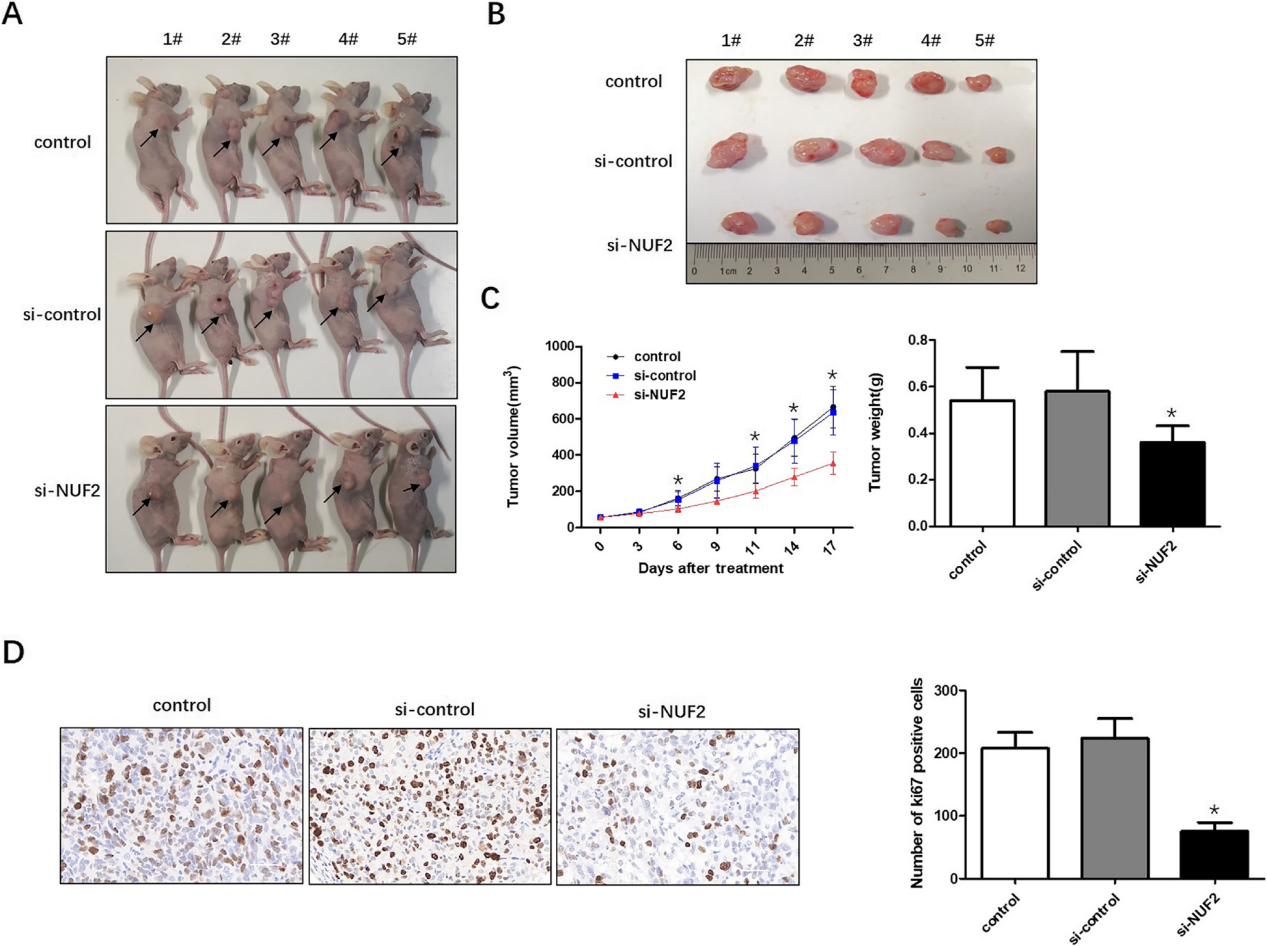
Silencing NUF2 inhibits OC progression in vivo. **A** SKOV3 cells were subcutaneously injected into the right flanks of BALB/c-nu mice. Mice were treated with PBS, si-control or modified si-NUF2. The sacrificed nude mice were photographed at 17 days after transplantation. **B** Tumors were derived from the nude mice of each group. **C** Tumor volume and weight were measured. Values are expressed as the mean ± SE. 2-tailed Student t test was used to determine significance. **D** Representative IHC images of tumors from nude mice for Ki-67 expression. Histograms (right) showed the number of Ki-67 positive cells. Values are expressed as the mean ± SE. 2-tailed Student t test was used to determine significance. *P < 0.05

Xenograft tumor model was used to validate the role of NUF2 in OC progression in vivo. BALB/c-nu mice were injected with PBS, modified si-NUF2 or their associated control cells. Tumor growth was measured for 17 days. As shown in Fig. 4A-C, compared with the PBS (control) and negative control group (si-control), mice in the si-NUF2 injection group (si-NUF2) exhibited slower tumor growth rate (*P* < 0.05) and lower tumor weight at the endpoint. Next, IHC assessment of cell proliferation marker Ki67 in the xenograft tumors above was performed. We confirmed that Ki67 expression was markedly suppressed in the si-NUF2 group (*P* < 0.05, Fig. 4D). These results suggested that NUF2 played a critical role in promoting OC progression in vivo.

### HNRNPA2B1 physically associates with and regulate NUF2

**Fig. 5:**
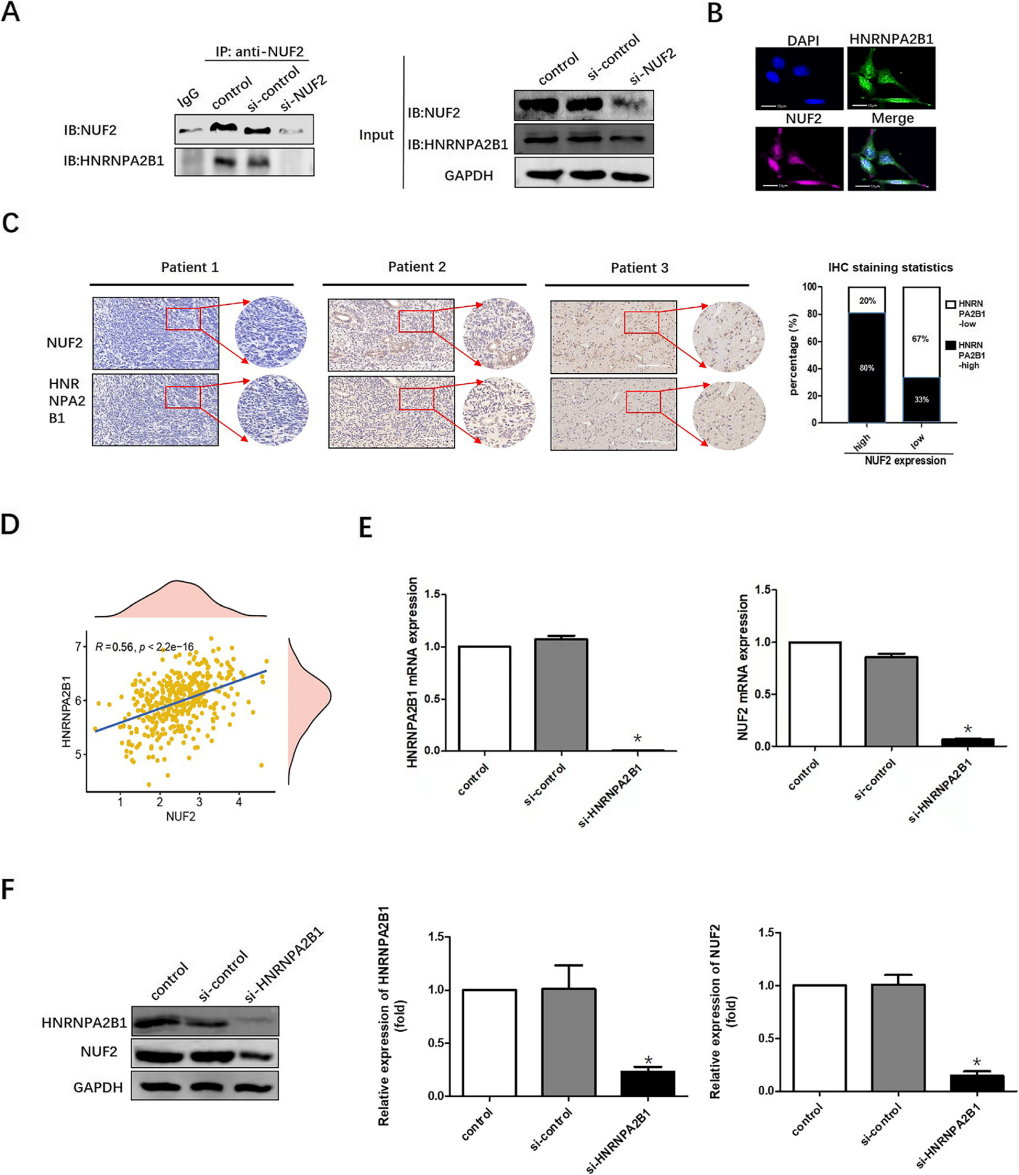
NUF2 physically associates with HNRNPA2B1. **A** IP assays were conducted in SKOV3 cells transfected with si-control; IgG was used as control. **B** Immunofluorescence staining showed that HNRNPA2B1 (green) co-localized with NUF2 (pink) in the SKOV3 cells. DAPI was used to stain the nuclear. Scale bars = 10 μm. **C** Representative IHC staining of HGSOC tumors for HNRNPA2B1 and NUF2 expression. Scale bars = 500 μm. IHC results revealed that expression of HNRNPA2B1 and NUF2 was positively interrelated in OC tissues as shown in the histogram (right). **D** GEPIA was applied to investigate the correlated expression between HNRNPA2B1 and NUF2. **E** Silencing HNRNPA2B1 decreased the mRNA expression of NUF2 by RT-qPCR assay. The experiment was repeated 3 times. In each experiment, HNRNPA2B1 or NUF2 mRNA levels were assessed by RT-qPCR and normalized to GAPDH. The HNRNPA2B1 or NUF2 mRNA levels were normalized to the control group that was arbitrarily set as 1.*P < 0.05. **F** Silencing HNRNPA2B1 decreased the protein expression of NUF2 by western blot. Quantification of HNRNPA2B1 and NUF2 expressions relative to GAPDH were shown. The experiment was repeated 3 times. In each time, the other groups were normalized to the control group that was arbitrarily set as 1. *P < 0.05

To investigate how NUF2 exert its functions in OC cells, potential NUF2 interacting proteins were analyzed using IP coupled with MS analyses. Proteomic analysis identified the proteins unique to the complexes related to NUF2 in the si-control group, whereas 74 proteins were found (Supplementary Fig. S1 and Supplementary Table 1). Among these, according to GEPIA, we identified HNRNPA2B1 as an NUF2-interacting protein. Next, we validated the relationship between NUF2 and HNRNPA2B1 by IP, immunofluorescence staining and IHC staining. HNRNPA2B1 was immunoprecipitated by the NUF2 antibody in si-control OC cells and control OC cells (Fig. 5A). Immunofluorescence staining showed that NUF2 and HNRNPA2B1 co-localized in OC cells (Fig. 5B). Moreover, we performed IHC staining of the tumors from 13 HGSOC patients and calculated the staining score of each slide. The IHC results indicated that about 67% of the tumor tissues (2 of 3) with low NUF2 expression showed negative or weak HNRNPA2B1 staining and 80% (8 of 10) of those with high NUF2 expression displayed moderate or strong HNRNPA2B1 staining (Fig. 5C). Besides, as shown in Fig. 5D, GEPIA analysis revealed a significant positive relationship between NUF2 and HNRNPA2B1 genes, and knockdown HNRNPA2B1 significantly decreased the mRNA and protein expression levels of NUF2 (Fig. 5E-F). Collectively, these results above indicated that HNRNPA2B1 physically interacted with and regulated NUF2.

### HNRNPA2B1/NUF2 activates the PI3K/AKT/mTOR signaling pathway

**Fig. 6:**
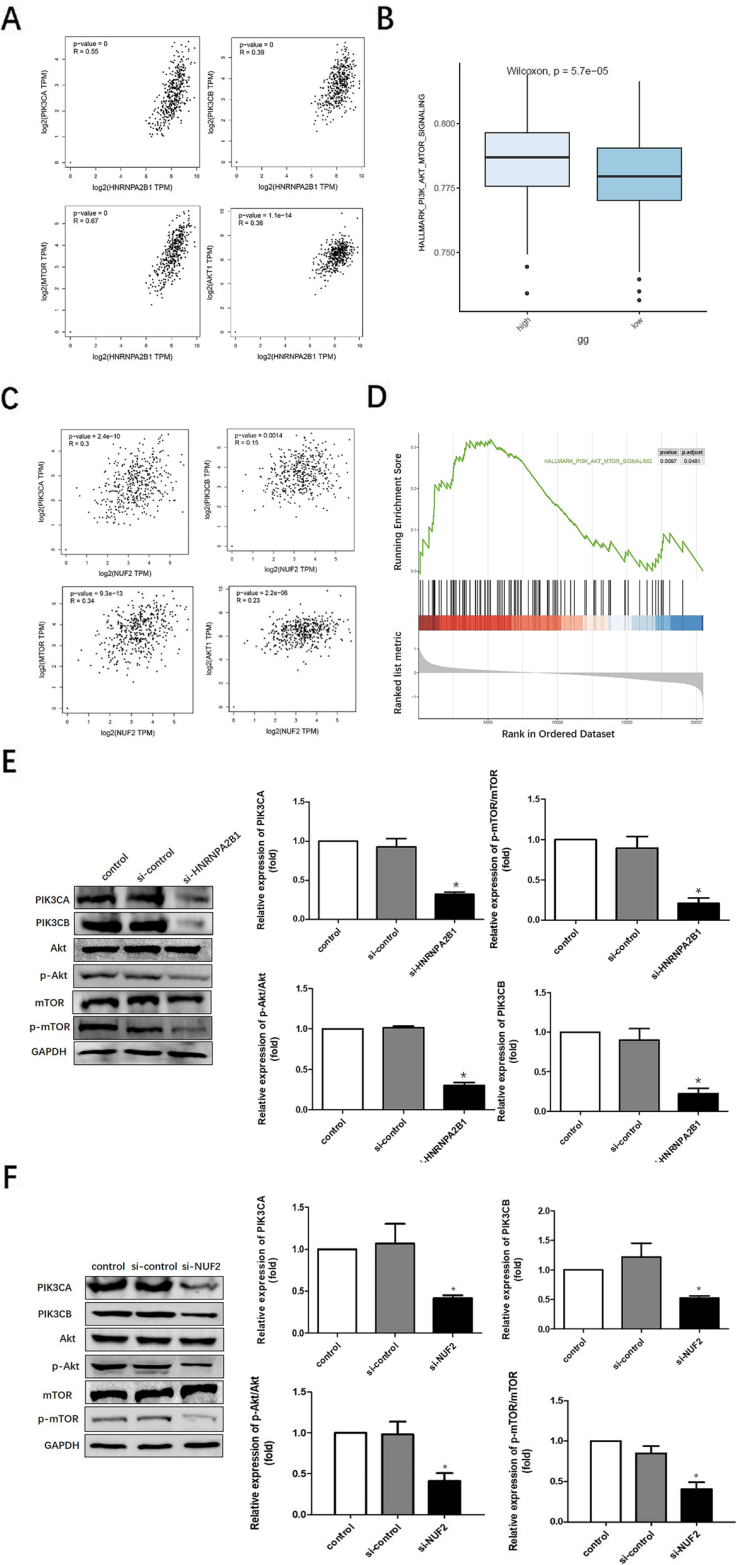
HNRNPA2B1-NUF2 active the PI3K/AKT/mTOR signaling pathway. **A** Scatter plots of HNRNPA2B1 and genes involved in PI3K/AKT/mTOR signaling pathways. **B** GSEA of enriched pathways in 180 samples exhibited high HNRNPA2B1 expression and in the remaining 180 samples exhibited low HNRNPA2B1 expression, which indicated that the high HNRNPA2B1 expression was related to PI3K/AKT/mTOR signaling pathway. *P* < 0.05. **C** Scatter plots of NUF2 and genes involved in PI3K/AKT/mTOR signaling pathways. **D** Enriched pathway found by the GSEA using MsigDB. PI3K/AKT/mTOR signaling pathway is enriched in OC samples with highly expressed NUF2. *P* < 0.05. **E**, **F** Western blot was conducted to analyze the expressions of PI3K/AKT/mTOR signaling pathway-related genes. The result was obtained by 3 independent experiments. In each experiment, the protein levels of PIK3CA or PIK3CB relative to GAPDH were normalized to the control group that was arbitrarily set as 1. The protein levels of p-AKT or p-mTOR related to the total AKT or mTOR were normalized to the control group that was arbitrarily set as 1. HNRNPA2B1 or NUF2 knockdown reduced the expression levels of PIK3CA, PIK3CB, p-AKT and p-mTOR. *P < 0.05

PI3K/AKT/mTOR signaling pathway plays an important role in cell proliferation and apoptosis of ovarian cancer cells [15]. Pathway enrichment analysis by GEPIA, GSEA and GSVA revealed a positive correlation between HNRNPA2B1 and PI3K/AKT/mTOR signaling pathway (*P* < 0.001, Fig. 6A-B). We also identified NUF2 involved in the PI3K/AKT/mTOR signaling pathways by GEPIA, GSEA and GSVA (*P* < 0.001, Fig. 6C-D). Hence, we tried to further explore the effects of HNRNPA2B1 or NUF2 expression on the PI3K/AKT/mTOR signaling pathway. SKOV3 cells were pretreated with HNRNPA2B1 siRNA or NUF2 siRNA and control siRNA respectively, and the expression of PI3K/AKT/mTOR signaling pathway-related molecules were examined by western blot. As shown in Fig. 6E-F, HNRNPA2B1 or NUF2 depletion decreased PIK3CA, PIK3CB, p-mTOR, p-AKT expression. These data demonstrated that oncogenic HNRNPA2B1 acted as an NUF2-interacting protein via PI3K/AKT/mTOR signaling pathway in HGSOC tumorigenesis.

## Discussion

OC is the first leading cause of cancer death among gynecological malignancies [16]. Accumulating evidence indicates that NUF2 plays a very important role in the progression of kinds of malignant tumors. This study aimed to explore the effect of NUF2 on progression of OC. We found that NUF2 expression was significantly upregulated in OC tumor tissues and OC cells, and might function as a critical prognostic indicator for OC patients. Silencing NUF2 inhibited proliferation, induced apoptosis of OC cells in vitro and blocked tumor growth in vivo. Furthermore, our results also demonstrated that oncogenic NUF2 functions as a tumor activator in OC progression by interacting with HNRNPA2B1 via the PI3K/AKT/mTOR signaling pathway.

According to literature reports, the cell division cycle-related protein family is necessary for the maintenance of normal cell functions, and its abnormal expression can promote the occurrence of tumors, and play an important role in regulating tumor cell development, invasion and metastasis, etc. [17]. As an important member of the cell division cycle-related protein family, NUF2 plays an important role in the correct attachment of kinetochore-microtubules and the normal separation of chromosomes during the process of cell mitosis. The wrong separation of chromosomes can lead to gene instability and ultimately induce tumor. Evidences were obtained regarding the roles of NUF2 in the progression of different kinds of cancers. In prostate cancer, Wong et.al found that knockdown NUF2 could significantly inhibit the growth of cancer cells [6]. Zhang et al. found that NUF2 acted as oncogenes in endometrial carcinoma cancer cells [18]. In colorectal cancer, studies have found that heterogeneous nuclear ribonucleoprotein K activated the transcription of NUF2, knockdown of NUF2 expression could significantly inhibit proliferation of colorectal cancer cells [8]. In melanoma tumors, NUF2 was highly expressed and significantly related to the poor prognosis of patients [19]. It was also found that poor breast cancer patient survival was significantly associated with high NUF2 expression caused by low TTP/HuR mRNA ratios [10]. In lung cancer, Chen showed that upregulated NUF2 gene expression level probably played a crucial part in oncogenesis, and could be used as the potential prognostic marker to improve survival rate of the patients [20]. In hepatocellular cancer, Xie found that high expression of NUF2 was correlated with poor survival and differential immune cell infiltration in patients [7]. Besides, another study suggested that NUF2 might modulate cell proliferation via cell cycle control in Osteosarcoma Saos-2 cells, depletion-induced growth inhibition was associated with cell cycle arrest and apoptosis [21]. However, the role of NUF2 in OC had not been specifically investigated. Based on bioinformatic analysis, Sethi et al. demonstrated that NUF2 might take part in the development of OC [22]. In this study, for the first time, we found that high NUF2 expression was related to the poor prognosis of OC patients. Moreover, NUF2 could increase the proliferation and decrease the apoptosis of OC cells. We also showed NUF2 silencing in mice harboring OC xenograft tumors induced a great reduction in tumor size and markedly suppressed levels of Ki-67.

Studies on NUF2 have so far primarily focused on its functions in tumorigenesis whereas the proteins interact with itself and the detailed mechanisms are still unclear. Increasing evidences showed that proteins of heterogeneous nuclear ribonucleoproteins family were closely associated with NUF2 in multiple cancers [6, 8]. Based on IP/MS associated with bioinformatics analysis, we herein found that HNRNPA2B1, which was reported highly expressed in OC cells and promoted the proliferation of OC cells [12], appeared to interact with NUF2. Furthermore, immunofluorescence staining and IHC staining showed a positive correlation between NUF2 and HNRNPA2B1. In addition, we found that HNRNPA2B1 could promote NUF2 protein translation, leading to OC carcinogenesis. Our findings indicated that the HNRNPA2B1-NUF2 expression likely acted as an important oncogenic driver in OC progression.

The development of malignant tumors are the results of interactions between various signal pathways. The phosphoinositol 3 kinase (PI3K)/ protein kinase B (AKT)/mammalian target of rapamycin (mTOR) signaling pathway is an important signal transduction pathway in regulating the proliferation and apoptosis of cancer cells by participating in cell cycle processes, producing precursor apoptotic proteins, controlling angiogenesis and promoting cancer cell invasion and metastasis [21]. Recent studies had indicated that the PI3K/AKT/mTOR signal pathway was highly mutated and hyper-activated in a majority of OC patients, and are associated with advanced grade and stage disease [23–25]. In our study, bioinformatic analysis suggested that HNRNPA2B1 or NUF2 participated in the PI3K/AKT/mTOR signaling pathway in OC. Furthermore, we proved that the expressions of PIK3A, PIK3B, p-AKT and p-mTOR were decreased in HNRNPA2B1-siRNA or NUF2-siRNA treated SKOV3 cells when compared with controls. These results demonstrated that PI3K/AKT/mTOR signaling pathway was the key signaling pathway involved in the regulation of the tumorigenesis by HNRNPA2B1 or NUF2 in OC. Taken together, these findings suggested that NUF2 might shed light on a novel therapeutic strategy for OC. Further research is required to verify the mechanisms underlying the interaction between NUF2 and HNRNPA2B1 for OC diagnosis, prognosis, or treatment.

## Supplementary Information

**Additional file 1: Figure S1.Table S2.** All the datasets used in the manuscript.

**Additional file 2.**

**Additional file 3.**

**Additional file 4.**
