# Supplementary material for: NUF2 promotes tumorigenesis by interacting with HNRNPA2B1 via PI3K/AKT/mTOR pathway in ovarian cancer
Source: J Ovarian Res. 2023 Jan 20;16:17. doi: 10.1186/s13048-023-01101-9 (PMC9862784; doi:10.1186/s13048-023-01101-9)
Supplement: Supplementary file 1 — Additional file 1: Figure S1.Table S2. All the datasets used in the manuscript. [file 13048_2023_1101_MOESM1_ESM.docx]

**Supplementary Information**

**Figure S1**


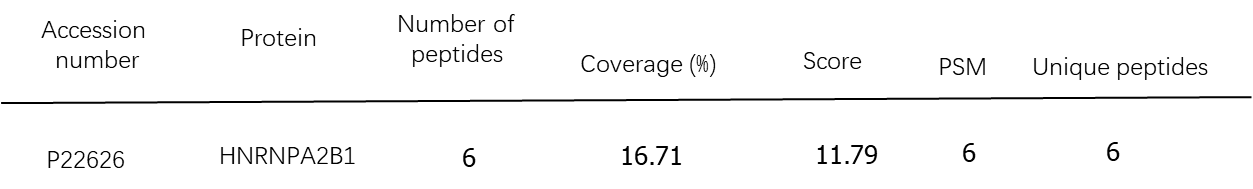


**Table S2**. All the datasets used in the manuscript.

1. The Cancer Genome Atlas (TCGA) OC datasets

| Data | Samples | Download |
| --- | --- | --- |
| RNA-seq | 309 | UCSC Xena (https://xenabrowser.net/datapages/) |

1. Kaplan-Meier plotter OC datasets

| Samples | Survival | Download |
| --- | --- | --- |
| 1436 | PFS | http://www.kmplot.com |
| 1657 | OS | http://www.kmplot.com |

1. GEPIA OC datasets

| Samples | Download |
| --- | --- |
| 426 | http://gepia.cancer-pku.cn |

1. Oncomine datasets

| Data | Samples | Download |
| --- | --- | --- |
| Lu Ovarian cancer | 5 normal samples and 45 tumor samples | http://www.oncomine.org |
| Yoshihara Ovarian cancer | 9 normal samples and 43 tumor samples | http://www.oncomine.org |
|  |  |  |
